# An Unaccounted Fraction of Marine Biogenic CaCO_3_ Particles

**DOI:** 10.1371/journal.pone.0047887

**Published:** 2012-10-23

**Authors:** Mikal Heldal, Svein Norland, Egil S. Erichsen, T. Frede Thingstad, Gunnar Bratbak

**Affiliations:** 1 Department of Biology, University of Bergen, Bergen, Norway; 2 Laboratory for Electron Microscopy, University of Bergen, Bergen, Norway; Instituto de Biologia, Brazil

## Abstract

Biogenic production and sedimentation of calcium carbonate in the ocean, referred to as the carbonate pump, has profound implications for the ocean carbon cycle, and relate both to global climate, ocean acidification and the geological past. In marine pelagic environments coccolithophores, foraminifera and pteropods have been considered the main calcifying organisms. Here, we document the presence of an abundant, previously unaccounted fraction of marine calcium carbonate particles in seawater, presumably formed by bacteria or in relation to extracellular polymeric substances. The particles occur in a variety of different morphologies, in a size range from <1 to >100 µm, and in a typical concentration of 10^4^–10^5^ particles L^−1^ (size range counted 1–100 µm). Quantitative estimates of annual averages suggests that the pure calcium particles we counted in the 1–100 µm size range account for 2–4 times more CaCO_3_ than the dominating coccolithophoride *Emiliania huxleyi* and for 21% of the total concentration of particulate calcium. Due to their high density, we hypothesize that the particles sediment rapidly, and therefore contribute significantly to the export of carbon and alkalinity from surface waters. The biological and environmental factors affecting the formation of these particles and possible impact of this process on global atmospheric CO_2_ remains to be investigated.

## Introduction

Calcification increases the *p*CO_2_ and acidity of seawater and is a central process in ocean-atmosphere CO_2_ exchange and in regulation of the global atmospheric CO_2_ level [1]–[3].

A large fraction of the carbon exported from the sea surface to deep waters and sediments is in the form of biogenic calcium carbonate (CaCO_3_) [4]. The importance of biogenic calcification is, in a geological perspective, evident from the global presence of calcium carbonate rocks like limestone and dolomite [5]. The relative contributions of the major planktonic CaCO_3_ producers, coccolithophores, foraminifera and pteropods, to global calcification rates are poorly constrained [4]. Coccolithophores and foraminifera have been estimated to account for 20–80% and 23–56% of biogenic carbonate exported from the photic zone [6], [7]. Pteropods and calcareous dinophytes are thought to be of minor importance and have been reported to account for <10% and 3.5% of the total planktonic carbonate production [6]. Precipitation of calcium carbonate particles induced by photosynthetic picoplankton and creating patches of particles (lime mud) called whiting event, are known to be of local or regional importance in both freshwater and tropical to subtropical marine environments [8], [9]. Carbonate precipitation is however also a common property of many different bacteria [10] and it is found to take place in many different aquatic and soil environments [11]–[13]. As far as we are aware, CaCO_3_ precipitation by marine bacteria was first described about 100 years ago [14] and later studied by a number of others [15]–[19]. The quantitative significance of the process *in situ* in marine waters has however remained undecided [10] and bacteria have hence not been considered among the major planktonic CaCO_3_ producers.

In this study we used X-ray fluorescence (XRF) to quantify total particulate calcium in seawater and analytical scanning electron microscopy (SEM) to observe, determine elemental composition of and count marine calcium particles harvested onto polycarbonate filters (pore size 0.6 or 1.0 µm).

## Materials and Methods

### Ethics Statement

No specific permits were required for the described field studies.

### Sampling

Raunefjorden (60^o^16.2′N, 5^o^12.5′E): Samples were collected from 5 m depth in Raunefjorden a coastal sampling station south of Bergen, Norway. CTD and chl *a* data were obtained with an STD SAIV a/s SD 204 with a Sea Point fluorometer (SAIV A/S, Environmental Sensors and Systems).

The Norwegian Sea: Samples were collected at 7 stations in The Norwegian Sea from, November 3.-8. 2010 during Cruise no 2010115 with R/V G.O. Sars. Samples were collected between 0 and 2500 m depth. CTD and chl *a* data were obtained with a Sea-Bird SBE 9. For details on sampling positions and depths see Fig. S1 and Table S1.

Bay Villefranche sur Mer, Point B (43°41.10′N–7°18.94′E): Samples were collected at 0, 10, 20, 30, 50 and 75 m on November 23. 2010. CTD and Chla *a* fluorescence data, obtained with a Seabird SBE 25, was provided by Service d’Observation en Milieu Littoral, INSU-CNRS, Point B, and by Service d’Observation de la Rade de Villefranche.

Kings Bay, Svalbard (78°58′N - 11°50E): Samples were collected at 5 m on June 14. 2010.

Sediment samples from the Norwegian continental margin: Sediment cores were collected in 1998 at 63°45'44''N; 05°15'19''E where the water depth is 840 m. The particles were sub sampled from the cores at ca 9 cm depth in the sediment, a stratum dated to 1953. (For details on cores see [20]).

Samples for counting of particles and X-ray analysis was prepared as described below within 1–4 h after sampling.

### Counting and X-ray Analysis of Particles in Scanning Electron Microscope

Particles from 200–750 mL of each water sample were harvested on a 0.6 µm (Raunefjorden and The Norwegian Sea) or 1 µm (Bay Villefranche sur Mer, Point B) pore size (25 mm diameter) polycarbonate membrane filter (GE Water & Process Technologies, PA, USA) within 2 h after sampling. Sediment samples for qualitative scrutiny were collected from the cores, resuspended in distilled water and filtered onto 1 µm filters as described above to make microscope specimens with a particle density appropriate for visual inspection.No fixatives, washing or staining was applied. The filters were air dried and coated for 30 seconds with Au/Pd in a Polaron SC502 Sputter Coater. The samples were viewed and analyzed in Zeiss Supra 55 VP scanning electron microscope equipped with EDS detector for X-ray elemental analysis. The microscope was operated at 8 kV accelerating voltage, and working distance of 4 mm. For X-ray microanalysis the instrument was operated at 15 kV accelerating voltage and working distance of 8–12 mm. Given the filter area, counting at 4000× magnification, known screen size and known sample volume, we calculated cells and particles pr mL.

The amount of CaCO_3_ in the particles we counted was estimated from particle abundance and volume, and assuming them to have a density of 2.7 g cm^−3^ as for calcite. The irregular morphology and variable size of the particles precluded accurate volume estimates based on microscope sizing. A conservative estimate of volume was nevertheless obtained assuming them to be spherical and with a mean diameter of 5 µm.The amount of CaCO_3_ in *E. huxleyi* was estimated from cell abundance, assuming a Ca content of 0.7 pg Ca pr coccolith and an average of 15 coccoliths pr cell [21].

### Quantification of Total Particulate Ca by X-ray Fluorescence

Particles from 0.5–2L of the water samples were harvested on filters within 2–4 h after sampling. The samples from Raunefjorden and from Bay Villefranche sur Mer, Point B were harvested onto 0.6 µm pore size (47 mm diameter) polycarbonate membrane filter (GE Water & Process Technologies, PA, USA). The samples from The Norwegian Sea were fractionated on 0.2, 1, 5 and 10 µm pore size (47 mm diameter) polycarbonate membrane filter (GE Water & Process Technologies, PA, USA) and the results added up to give particulate Ca>0.2 µm or on 0.2 µm pore size (47 mm diameter) Anodisc Al filters (Whatman, Maidstone, UK).

The filters were rinsed briefly by pulling 5 mL deionized water through the filters after filtration of the samples to remove seawater ions and reduce background signal. Excess water on the back and the brim of the filters was blotted off using clean paper tissue and they were then air dried at room temperature before analyses. Analyzing filters with and without rinsing with deionized water showed that the procedure did not affect the Ca measurements. The filters were analyzed in a Bruker AXE S4 Pioneer WDXRF instrument. The Ca measurements were calibrated with CaCl_2_ dissolved in ethanol to known concentration, applied to a silver plate and air dried before analysis.

## Results and Discussion

**Figure 1:**
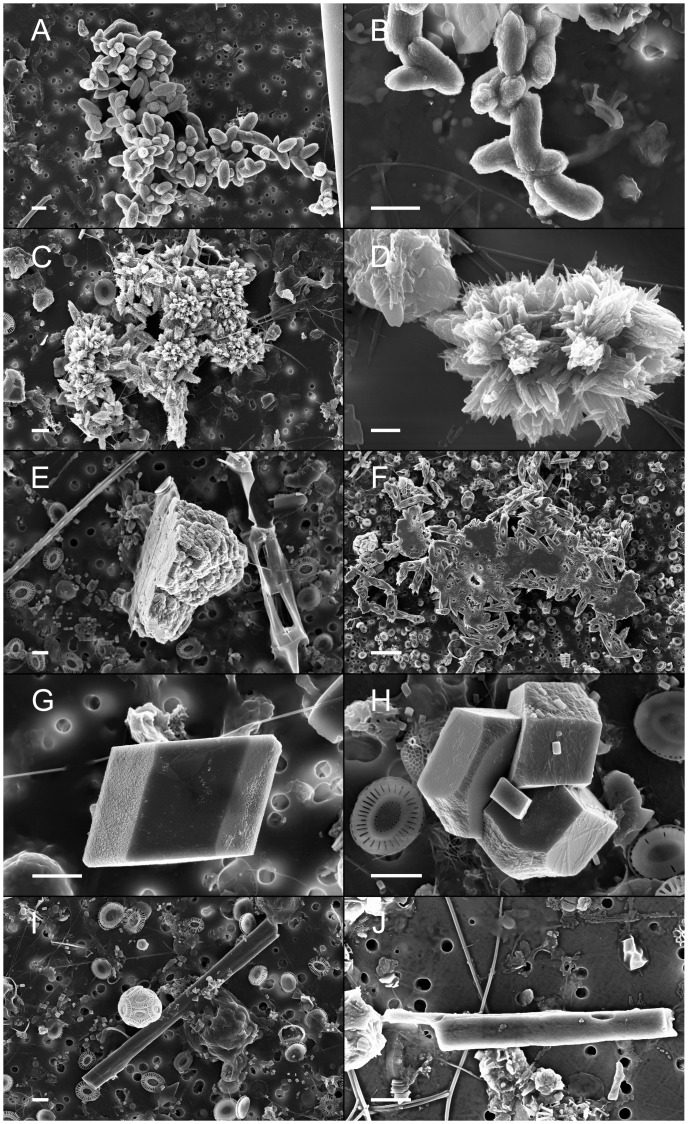
Scanning electron microscope images of marine calcium particles with different morphology. Samples were collected at 5 m depth in Raunefjorden, a coastal sampling station south of Bergen, Norway. A and B) Particles resembling bacteria and microcolonies of bacteria. B and D) Particles similar to the Ca carbonates described to precipitate on the cell surface of cultured marine bacteria. E and F) Particles with one flat surface suggesting that they are formed on a surface or interface. G and H) Particles with rhombohedral shape. I and J) Baton like particles resembling Bahaman ooids. All scale bars are 2 µm except in d) where it is 1 µm and f) where it is 10 µm.

The CaCO_3_ particles we observed and counted were of different morphology (Fig. 1). Some particles had a morphology that resembles bacteria or micro colonies of bacteria (Fig. 1a–b). Others (Fig. 1 c–d) were very similar to the Ca carbonates described to precipitate as needles and microcrystalline aragonite growing both parallel and perpendicular to the cell surface of cultured marine heterotrophic bacteria [22], or as the 20–30 µm long and 5–10 µm wide “Weisenkörner” found in Black Sea sediments [23]. Some particles had one flat surface (Fig. 1e–f) and resemble the highly oriented vaterite CaCO_3_ tablet-like arrays with a smooth air-side and rugged water-side formed at the air/water interface in the presence of polypeptides [24]. Particles with rhombohedral (Fig. 1 g–h) or baton like (Fig. 1 i–j) structure were also relatively frequent in our material. The former resembles calcite particles formed under experimental conditions in the presence of bacteria and bacterial extracellular polymers [25]. The latter show some resemblance to the baton like particles found in Bahaman ooids and interpreted as calcified rod-shaped bacteria [26]. The differences in morphology strongly suggest that the particles result from different processes or differences in the precipitation nuclei. We find it unlikely that the particles we have observed should originate from any land based mineral sources and since some have the appearance of growing crystals we conclude that they are formed *in situ*.

**Figure 2:**
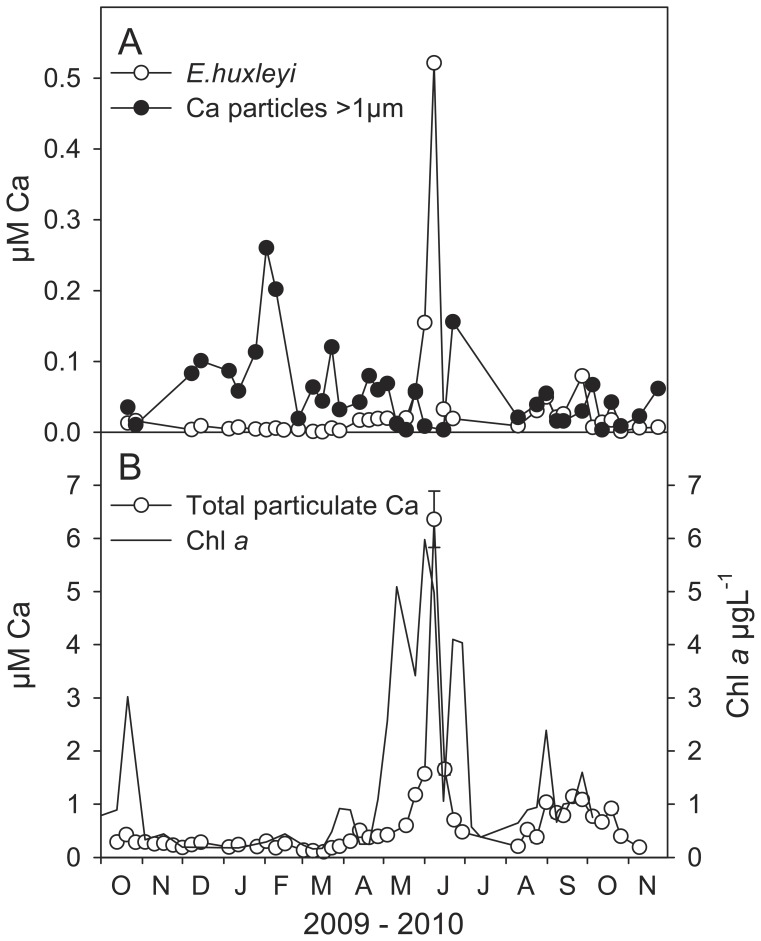
The annual variation in CaCO_3_ particles and chl *a.* Samples were collected at 5 m depth in Raunefjorden, a coastal sampling station south of Bergen, Norway. A) Calcium in the coccolithophore *Emiliania huxleyi* and in Ca particles estimated from scanning electron microscope counts. B) Total particulate Ca concentration measured by X-ray fluorescence (error bars are SE, n = 3–4) and chl *a* concentration. Hydrographical data and chl *a* profiles are shown in Fig. S4.

The microscope’s X-ray detector was used to assess the elemental composition of particles and to identify calcium containing particles before counting. The chemical composition of the particles varied from nearly pure CaCO_3_ to particles containing significant and variable amounts of Mg, Fe and Si (Fig. S2 and S3). In this context we counted the pure CaCO_3_ particles with size >1 µm. The annual variation in CaCO_3_ particles observed at 5 m depth in Raunefjorden, a coastal sampling station south of Bergen, Norway, is shown in Fig. 2a in comparison to CaCO_3_ in the coccoliths of *Emiliania huxleyi*. Hydrographical data and chl *a* profiles are shown in Fig. S4. The abundance of CaCO_3_ particles ranged from undetectable to 150 000 particles L^−1^ with an average of about 30 000 particles L^−1^. The abundance of *E. huxleyi* was typically in the range of 10^3^–10^5^ cells L^−1^ except for the bloom period when it reached 2 10^6^ cells L^−1^. The abundance of other coccolithophores like *Syracosphaera borealis, Algirosphaera robusta* and *Acanthoica quattrospina* never exceeded 5000 cells L^−1^.

The counting based estimates of CaCO_3_ concentration gave an annual average of 60±50 nM in particles and 30±80 nM in *E. huxleyi* (Mean±SD). Excluding the bloom, the annual average for *E. huxleyi* was 14±15 nM. The total concentration of particulate calcium, as measured by XRF analysis (Fig. 2b), amount to an annual average of 420±280 nM excluding the *E. huxleyi* bloom period with a peak value of 6400 nM when the abundance of free coccoliths was high. The XRF analysis will, in addition to the CaCO_3_ in the pure CaCO_3_ particles with size >1 µm we counted, also include the CaCO_3_ in smaller particles (0.2–1 µm), in free coccoliths, in faecal pellets, and in particles containing additional elements like Mg, Fe or Si. This suggests that our estimate of particulate CaCO_3_ based on counting of particles and *E. huxleyi* cells is conservative and that the CaCO_3_ particles, in comparison to coccolithophores may contribute significantly to the CaCO_3_ budget in surface waters. We note that there is a significant positive correlation (R = 0.644, p<0.0003, DF = 25) between total concentration of particulate calcium and chl *a* (Fig. 2b) suggesting that there is a link between CaCO_3_ particle production and biological production.

In samples collected along two transects across The Norwegian Sea (3–8. Nov. 2010) at 68–70°N, 5–14°E and at 63–64^o^N, 0–4^o^E and at depths from 5 to 2500 m (Fig. S1, Table S1) we found similar particles (Fig. S5) and a particulate Ca concentration of 220±100 nM (Mean±SD) (Fig. S6) but no obvious correlation with depth, distance offshore or chl *a.* Similar particles (Fig. S7) were also found in samples collected at 1–75 m depths in the Mediterranean Sea (Bay Villefranche sur Mer, Point B, 23. Nov. 2010) where the particulate Ca concentration was 430±120 nM (Mean±SD) (Fig. S8) and in samples collected at 5 m depth in Kings Bay (Svalbard), indicating a ubiquitous distribution in the ocean. Moreover, both amorphous particles and particles with one flat surface were found at ca 9 cm depth in sediment core samples collected at 840 m depth on the Norwegian continental margin (Fig. S9).

The formation of the CaCO_3_ particles we have observed may be related to bacterial activity and processes generating biogeochemical conditions facilitating precipitation [13], [27] or to chemical processes driving precipitation in bacterial outer structures such as exopolysaccharides and capsular polysaccharides [13], [28]. These conditions and processes may involve nucleation sites, pH, electrostatic charge, ionic concentration etc. and make the microenvironment in and on the cell wall and adjacent matrix different from the surrounding bulk habitat. The biofilm formed at the air-sea surface interface by coagulation and aggregation of dissolved organic substances released from phytoplankton bacteria and other organisms [29] is another possible site of calcium carbonate precipitation [13], [24]. The variety of different processes and chemical microenvironments possibly involved in precipitation and formation of CaCO_3_ particles may explain the morphological diversity of the CaCO_3_ particles [28]. The diversity may also be related to different bacteria precipitating different amounts, shapes, and types of carbonate crystals [28]. The CaCO_3_ particles we have observed may be classified as different morphotypes (Fig. 1) but each morphotype is far more variable in size and structure than the CaCO_3_ particles produced by e.g. coccolithophores and foraminifera, supporting the view that bacterial CaCO_3_ precipitation is not very carefully controlled or structurally directed [13]. Correlation between particulate calcium and chl *a* concentration suggests that biological processes are important but additional data such as bacterial biomass and production is not available to support this view. A comprehensive understanding of the mechanisms, conditions and (micro)environments facilitating CaCO_3_ precipitation in pelagic ecosystems is also lacking and precludes a more detailed interpretation of the results. The processes producing these particles are in any case different from the calcification processes currently considered to be of major importance, i.e. coccolithophores, foraminifera and pteropods [1], [6], [7], the environmental factors that regulate their production remains elusive and the subject calls for further studies.

Estimates of marine particulate CaCO_3_ concentration will include the particles we have described here when based on chemical analysis of particulate material collected by filtration. They may however not be included in estimates based on optical and remote sensing techniques presuming the particles to have optical properties that are different from those considered when calibrating and verifying these techniques. If the production of the particles takes place in the bulk water phase they are presumably also included in current estimates of calcification based on ^14^C–CO_2_ or ^45^Ca incorporation, but if the production is closely coupled to interfaces they may add to current production estimates. The flux of CaCO_3_ particles from surface waters to the deep ocean, and hence their role in ocean carbon cycle, depends on sedimentation and dissolution rates. The sedimentation rate of the pure calcium particles we have observed will, due to a higher density, presumably be higher than the sedimentation rate of similarly sized calcifying organisms like coccolithophores and foraminifera (<5− >100 µm) which in addition to calcite contains significant amounts of organic material. The crystal structure of the particles, aragonite being more soluble than calcite [30], and the absence of a protective organic coating [21] as for coccoliths and foraminifera tests, will influence the depth the particles may reach before they dissolve. Observations of similar particles in marine sediment samples [23], [31] calls for cautious interpretation but may suggest that particles produced in surface waters reach the seafloor at some depth and hence contribute to the export of carbon from surface waters. Moreover, bacterial calcification may provide an additional explanation for the discrepancy between coccolith-CaCO_3_ and total-CaCO_3_ flux estimates that has been noted in some sediment trap studies [32], [33].

The global carbonate budget is far from resolved [4], [34] and balancing production, sedimentation and dissolution processes on the global scale does therefore not exclude the possibility for significant additional sources of CaCO_3_ in ocean surface waters. The novel CaCO_3_ particles described and counted in this study amount to 21±28% (Mean±SD) of the total concentration of particulate calcium. Our estimates are crude but in concert with the ubiquitous distribution of the particles it suggests nonetheless that they may be of quantitative significance in the global carbonate budget. Calcium precipitation associated with bacterial activity appears to be a common process which also has been related to carbonate sediment formation [11], [35], [36]. Understanding the geological record of calcareous rocks and sediments is based on knowledge about the organisms considered to be the main producers of calcium carbonate (coccolithophores and foraminifera), including their occurrence, abundance, size, habitat requirements etc. If bacteria have been an additional quantitatively significant source of calcium carbonate in marine ecosystems it may call for a reexamination to affirm the current interpretation.

## Supporting Information

Figure S1 **Map showing sampling positions in The Norwegian Sea.** Station 649, 654, 658 and 664 are from the Gimsøy transect. Station 677, 668 and 667 are from the Svinøy transect. The positions of the sampling stations are given in Table S1.
(PDF)

Figure S2 **X-ray spectrum of a marine calcium carbonate particle.** X-ray spectrum of a pure calcium carbonate particle containing only carbon, oxygen and calcium. The peak at 2.12 keV is gold from the AuPd sputter coating of the samples. Non-pure calcium carbonate particles contained significant and variable amounts of Mg (K-line at 1.25 keV), Fe (K-line at 6.4keV and L-line at 0.7 keV) and Si (K-line at 1.74 keV).
(PDF)

Figure S3 **X-ray mapping of marine particles.** X-ray mapping of particles collected at 15 m depth in Bay Villefranche sur Mer, Point B, on November 23, 2010. Ca containing particles labelled red (left panel) and Si containing particles labelled green (right panel). Comparing the X-ray mappings with the greyscale image (centre) shows that some particles contain Ca and no Si and *vice versa*. Some amorphous particles contain both Ca and Si. The images demonstrate the difficulty of recognizing calcium carbonate particles by morphology alone.
(PDF)

Figure S4 **Hydrography and chl** ***a*** **profiles from Raunefjorden.** Isopleth plots of temperature, salinity, density and chl *a* concentration in Raunefjorden, a coastal sampling station south of Bergen, Norway, during the sampling program 2009–2010.
(PDF)

Figure S5 **Scanning electron microscope images of calcium carbonate particles from The Norwegian Sea.** Particles collected at A) Station 658, 50 m depth. B) Station 677, 100 m depth. C) Station 658. 5 m depth, D) Close up of the particle shown in C. For positions of sampling stations see Fig. S1 and Table S1.
(TIF)

Figure S6 **Hydrography, chl** ***a*** **and particulate Ca in the the Norwegian Sea.** Depths profiles from the Norwegian Sea, November 3.-8. 2010 showing water density (black line), salinity (blue line), temperature (red line), chl *a* concentration (green line) and particulate Ca concentration (black dots, error bar shows SEM, n = 3). The bottom is marked with a dotted line when different from the X-axis. For positions of sampling stations see Fig. S1 and Table S1.
(PDF)

Figure S7 **Scanning electron microscope images of calcium carbonate particles.** A and B) Particles from 1 m depth in The Mediterranean Sea, Bay Villefranche sur Mer, Point B. C) Particle from 5 m depth in Kings Bay (Svalbard).
(TIF)

Figure S8 **Hydrography, chl** ***a*** **and particulate Ca in the Mediterranean Sea.** Depths profile from Bay Villefranche sur Mer, Point B (43°41.10′N–7°18.94′E on November 23. 2010 showing water density (black line), salinity (blue line), temperature (red line), chl *a* fluorescence (green line) and particulate Ca concentration (black dots, error bar shows SEM, n = 3–4). CTD and chl *a* fluorescence data, obtained with a Seabird SBE 25, was provided by Service d’Observation en Milieu Littoral, INSU-CNRS, Point B, and by Service d’Observation de la Rade de Villefranche.
(PDF)

Figure S9 **Scanning electron microscope images of calcium carbonate particles in sediment core samples.** Sediment core samples were collected at 840 m depth on the Norwegian continental margin. The particles were sub sampled at ca 9 cm depth in the sediment cores, a stratum dated to 1953. Note the flat surface of the particle in A. For details on cores see [20].
(TIF)

Table S1 **Table of sampling times and positions in The Norwegian Sea, R/V G.O. Sars, Cruise no 2010115.**
(PDF)
