# Supplementary figures and images for: An Unaccounted Fraction of Marine Biogenic CaCO3 Particles
Source: PLoS One. 2012 Oct 23;7(10):e47887. doi: 10.1371/journal.pone.0047887 (PMC3479124; doi:10.1371/journal.pone.0047887)

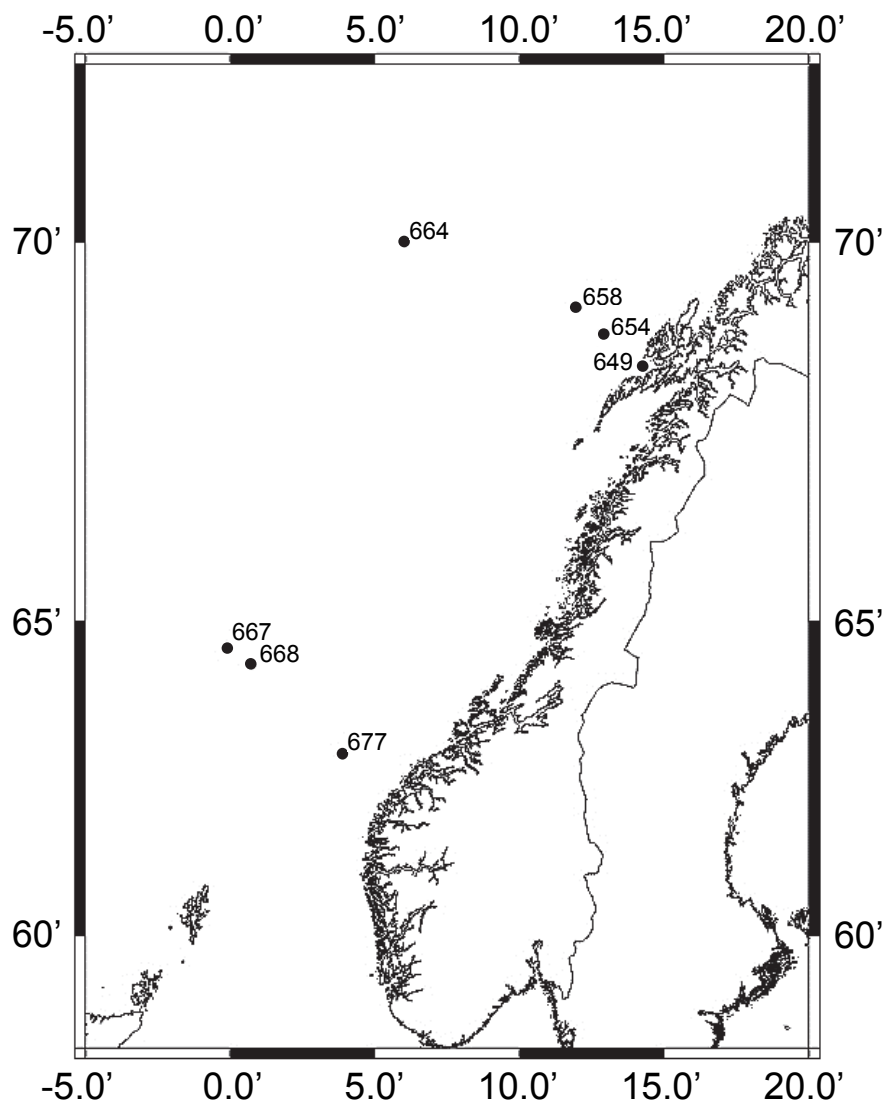

Supplement: Figure S1 — Map showing sampling positions in The Norwegian Sea. Station 649, 654, 658 and 664 are from the Gimsøy transect. Station 677, 668 and 667 are from the Svinøy transect. The positions of the sampling stations are given in Table S1. (PDF) [file pone.0047887.s001.pdf]

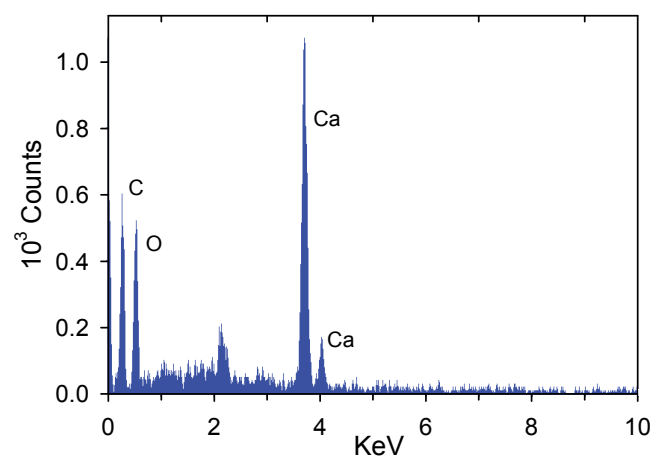

Supplement: Figure S2 — X-ray spectrum of a marine calcium carbonate particle. X-ray spectrum of a pure calcium carbonate particle containing only carbon, oxygen and calcium. The peak at 2.12 keV is gold from the AuPd sputter coating of the samples. Non-pure calcium carbonate particles contained significant and variable amounts of Mg (K-line at 1.25 keV), Fe (K-line at 6.4keV and L-line at 0.7 keV) and Si (K-line at 1.74 keV). (PDF) [file pone.0047887.s002.pdf]

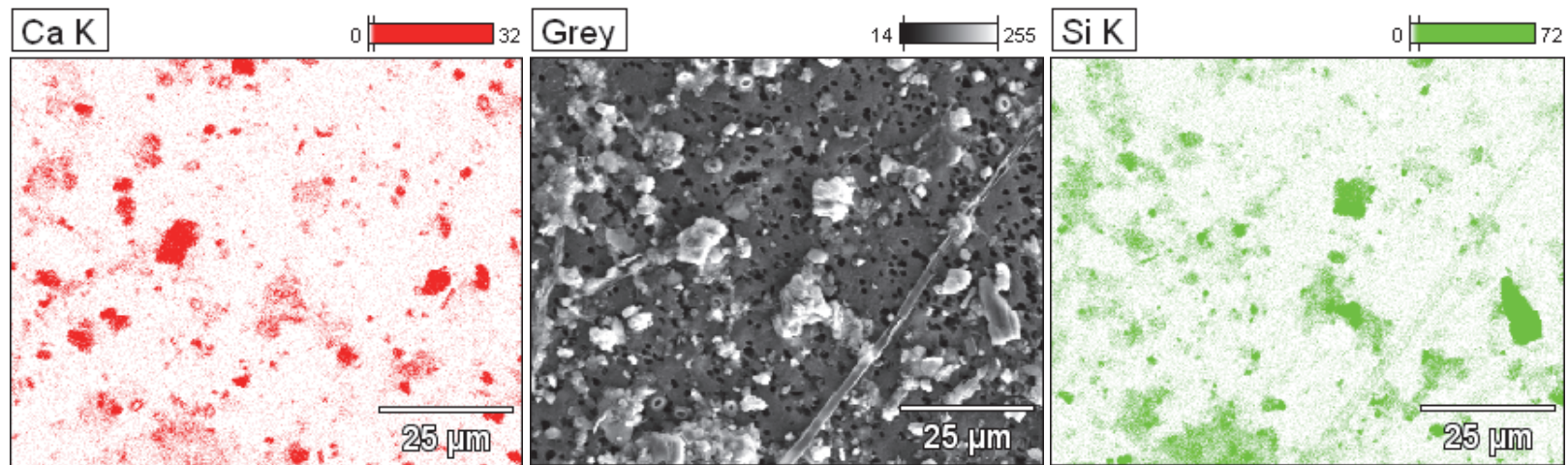

Supplement: Figure S3 — X-ray mapping of marine particles. X-ray mapping of particles collected at 15 m depth in Bay Villefranche sur Mer, Point B, on November 23, 2010. Ca containing particles labelled red (left panel) and Si containing particles labelled green (right panel). Comparing the X-ray mappings with the greyscale image (centre) shows that some particles contain Ca and no Si and vice versa. Some amorphous particles contain both Ca and Si. The images demonstrate the difficulty of recognizing calcium carbonate particles by morphology alone. (PDF) [file pone.0047887.s003.pdf]

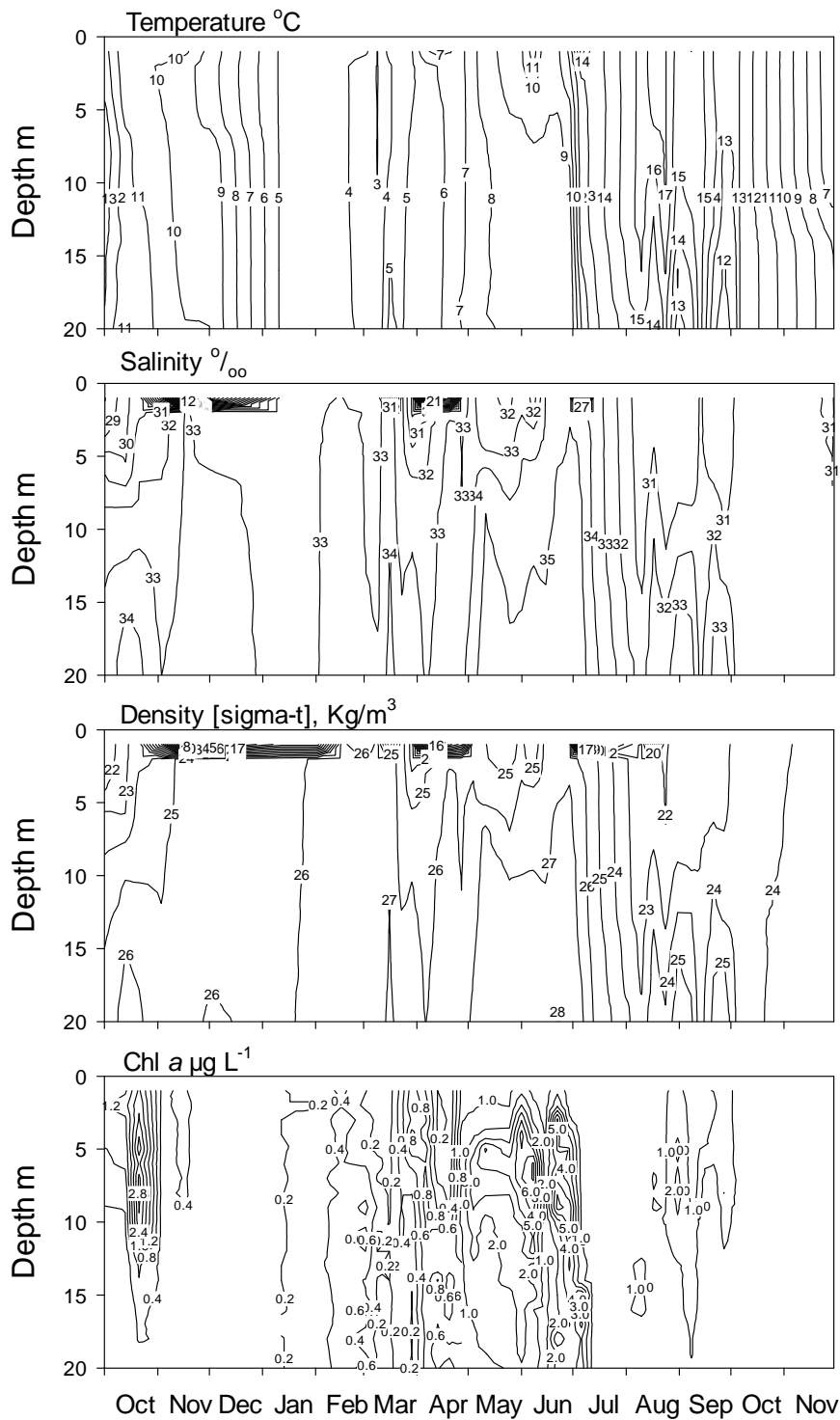

2009 - 2010

Supplement: Figure S4 — Hydrography and chl a profiles from Raunefjorden. Isopleth plots of temperature, salinity, density and chl a concentration in Raunefjorden, a coastal sampling station south of Bergen, Norway, during the sampling program 2009–2010. (PDF) [file pone.0047887.s004.pdf]

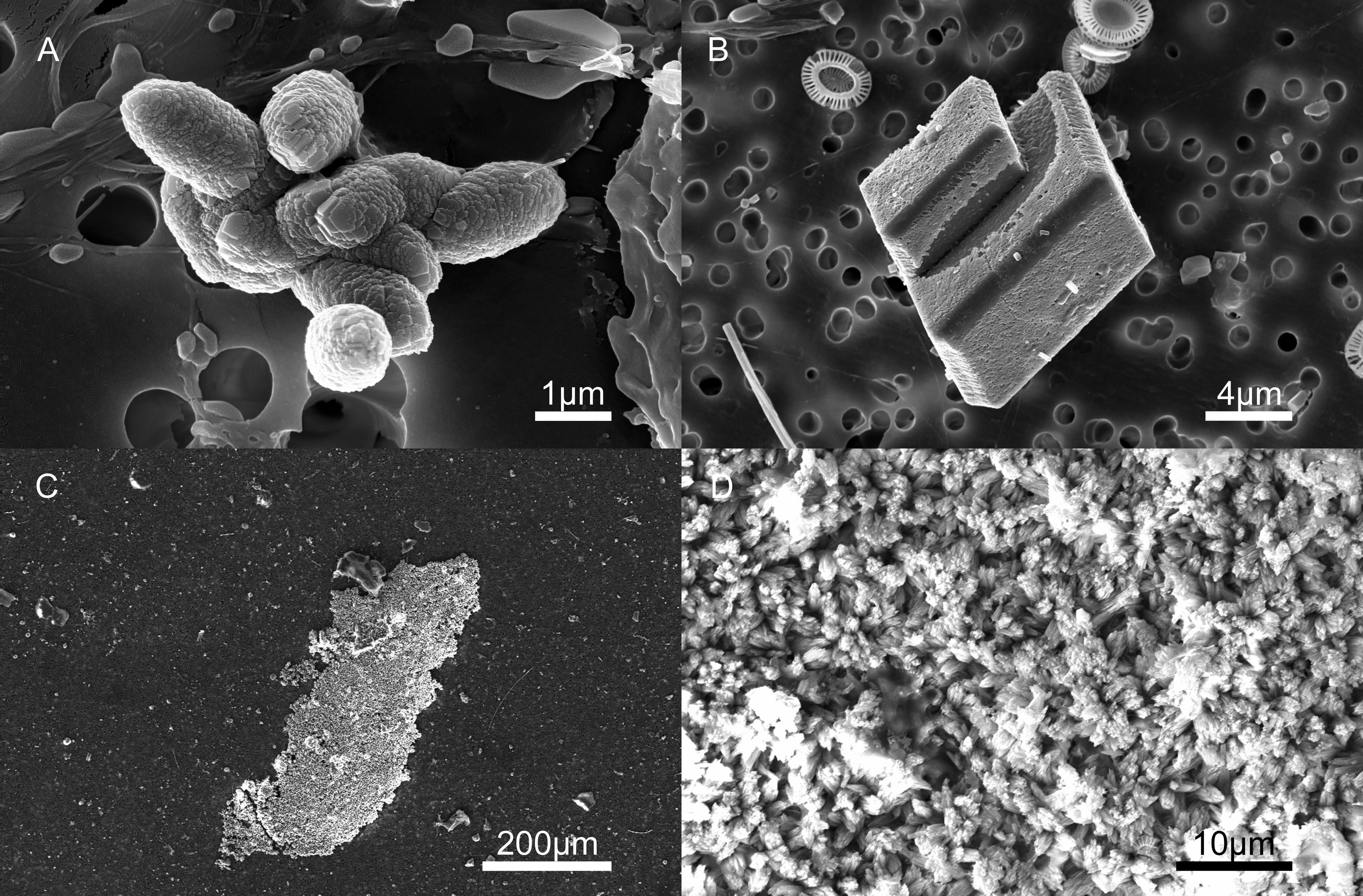

Supplement: Figure S5 — Scanning electron microscope images of calcium carbonate particles from The Norwegian Sea. Particles collected at A) Station 658, 50 m depth. B) Station 677, 100 m depth. C) Station 658. 5 m depth, D) Close up of the particle shown in C. For positions of sampling stations see Fig. S1 and Table S1. (TIF) [file pone.0047887.s005.tif]

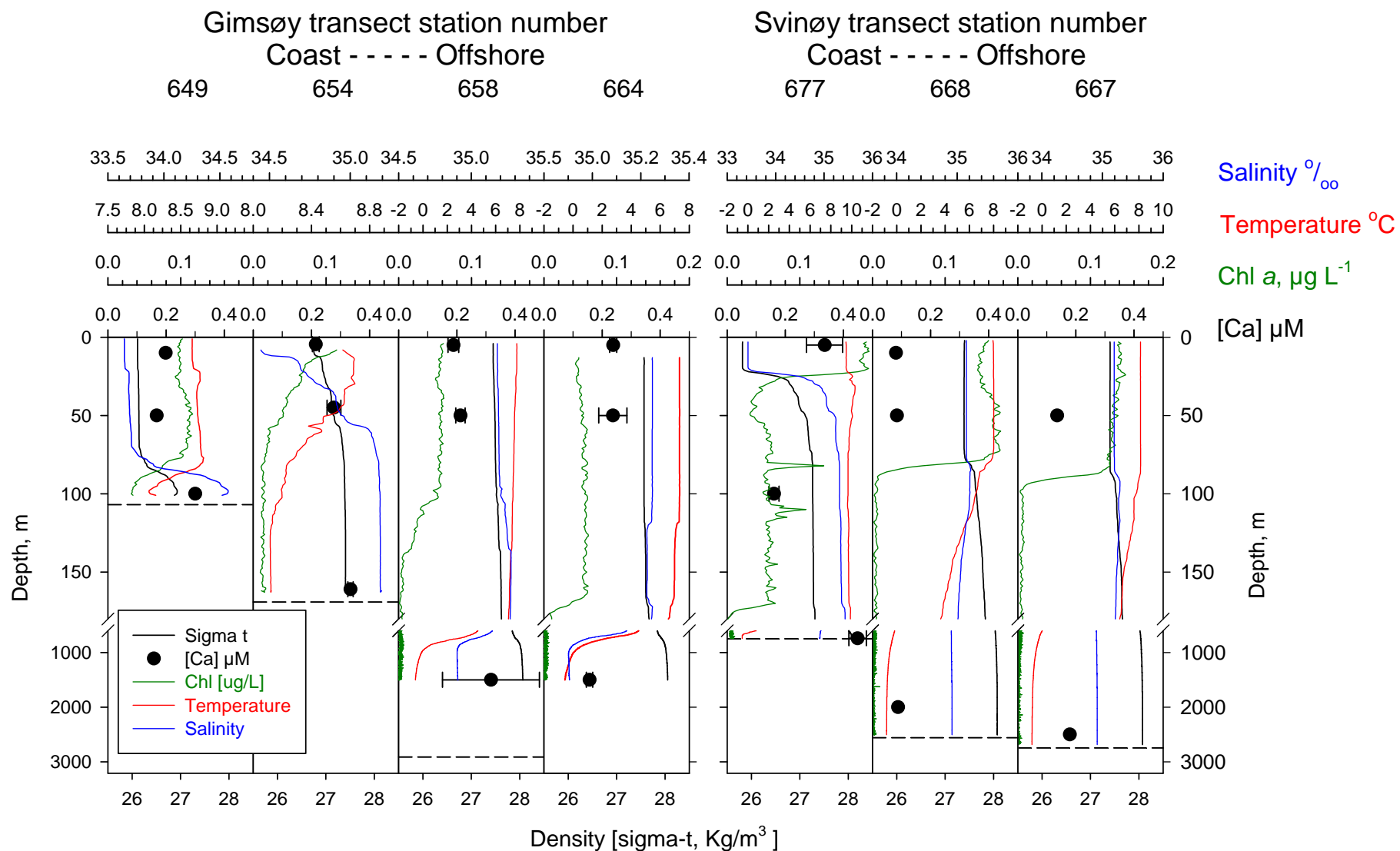

Supplement: Figure S6 — Hydrography, chl a and particulate Ca in the the Norwegian Sea. Depths profiles from the Norwegian Sea, November 3.-8. 2010 showing water density (black line), salinity (blue line), temperature (red line), chl a concentration (green line) and particulate Ca concentration (black dots, error bar shows SEM, n = 3). The bottom is marked with a dotted line when different from the X-axis. For positions of sampling stations see Fig. S1 and Table S1. (PDF) [file pone.0047887.s006.pdf]

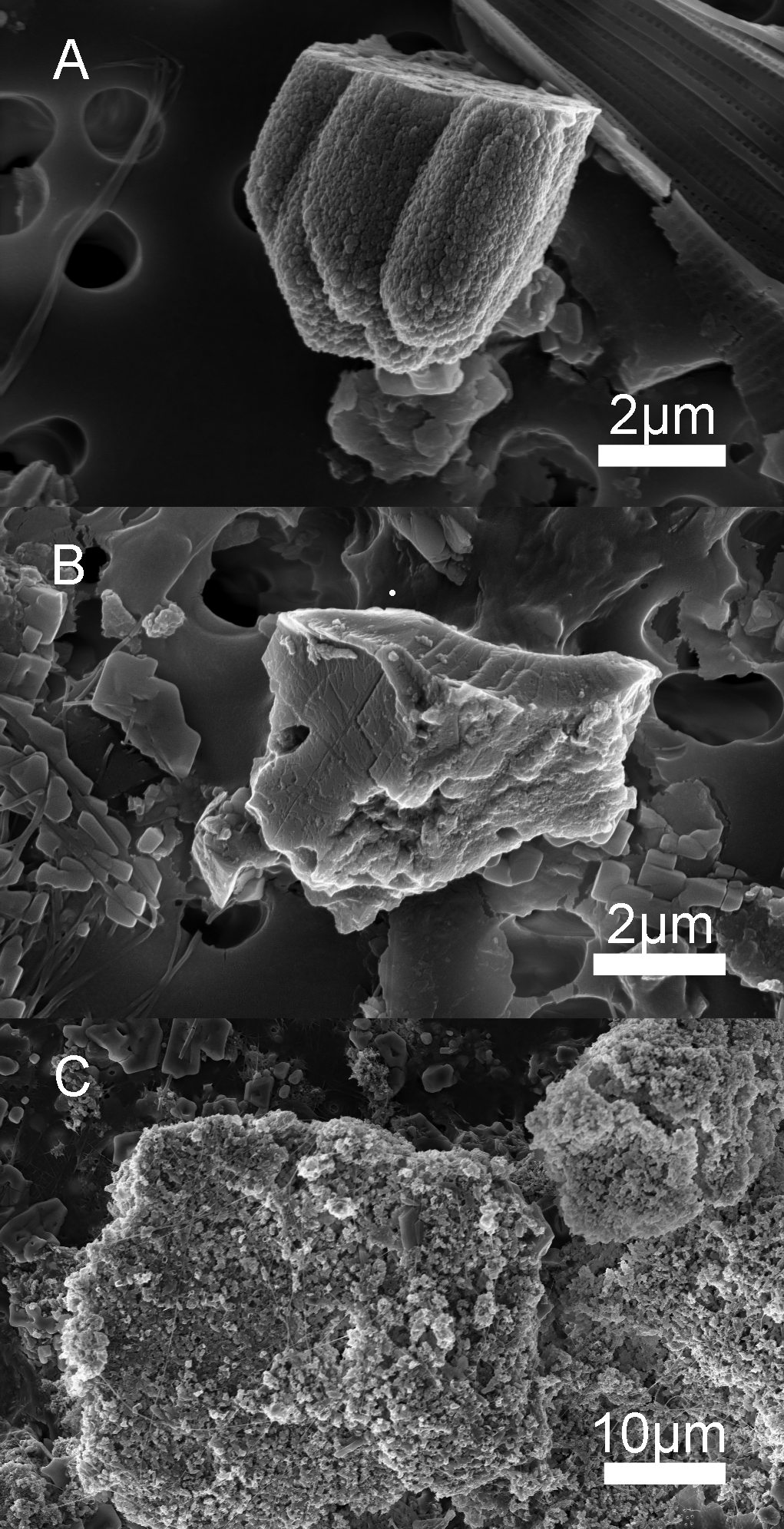

Supplement: Figure S7 — Scanning electron microscope images of calcium carbonate particles. A and B) Particles from 1 m depth in The Mediterranean Sea, Bay Villefranche sur Mer, Point B. C) Particle from 5 m depth in Kings Bay (Svalbard). (TIF) [file pone.0047887.s007.tif]

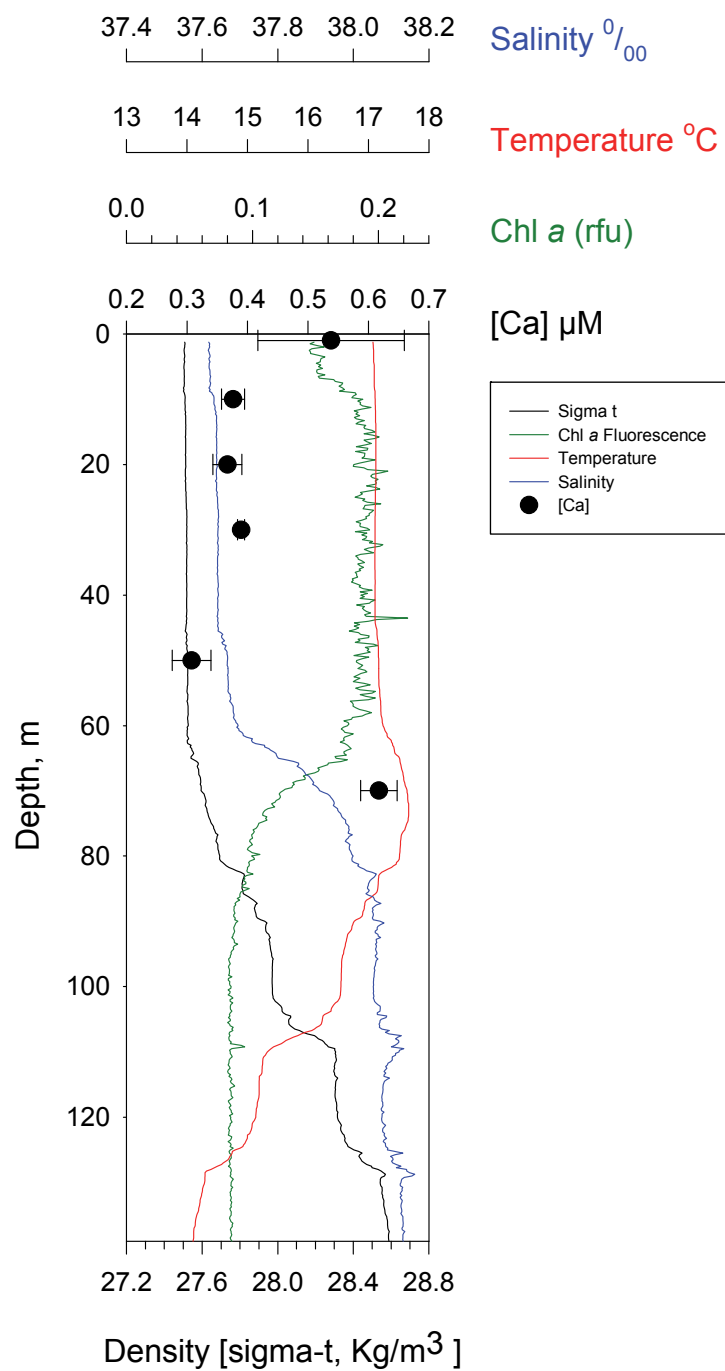

Supplement: Figure S8 — Hydrography, chl a and particulate Ca in the Mediterranean Sea. Depths profile from Bay Villefranche sur Mer, Point B (43°41.10′N–7°18.94′E on November 23. 2010 showing water density (black line), salinity (blue line), temperature (red line), chl a fluorescence (green line) and particulate Ca concentration (black dots, error bar shows SEM, n = 3–4). CTD and chl a fluorescence data, obtained with a Seabird SBE 25, was provided by Service d’Observation en Milieu Littoral, INSU-CNRS, Point B, and by Service d’Observation de la Rade de Villefranche. (PDF) [file pone.0047887.s008.pdf]

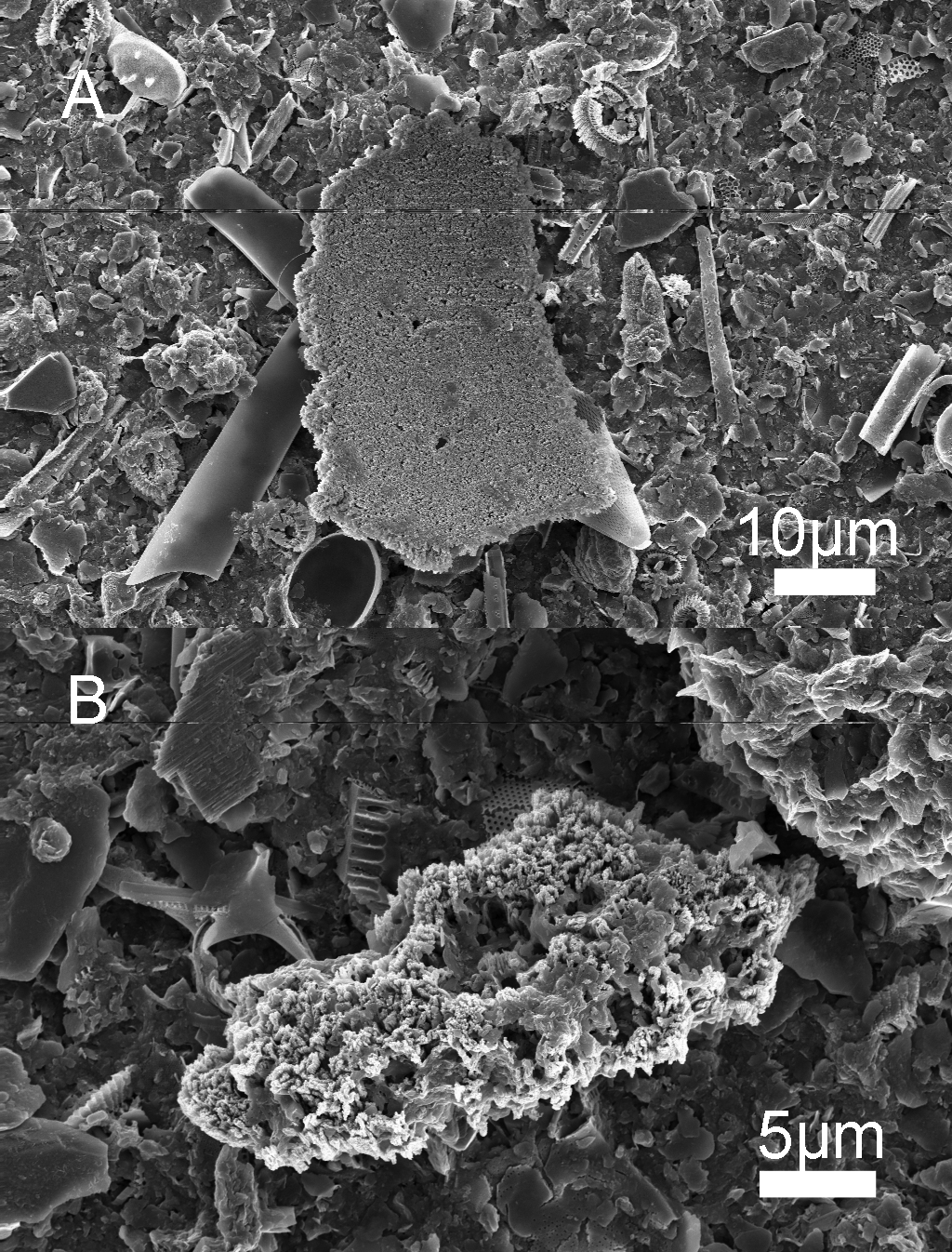

Supplement: Figure S9 — Scanning electron microscope images of calcium carbonate particles in sediment core samples. Sediment core samples were collected at 840 m depth on the Norwegian continental margin. The particles were sub sampled at ca 9 cm depth in the sediment cores, a stratum dated to 1953. Note the flat surface of the particle in A. For details on cores see [20]. (TIF) [file pone.0047887.s009.tif]
