# Supplementary material for: An Unaccounted Fraction of Marine Biogenic CaCO3 Particles
Source: PLoS One. 2012 Oct 23;7(10):e47887. doi: 10.1371/journal.pone.0047887 (PMC3479124; doi:10.1371/journal.pone.0047887)
Supplement: Table S1 — Table of sampling times and positions in The Norwegian Sea, R/V G.O. Sars, Cruise no 2010115. (PDF) [file pone.0047887.s010.pdf]

Table S1

Table of sampling times and positions in The Norwegian Sea, R/V G.O. Sars, Cruise no 2010115.

Station	Date	NMEA UTC (Time)	NMEA Latitude	NMEA Longitude	Echodepth (m)
sta0649	Nov 03 2010	5:47:21	68 25.70 N	014 00.79 E	107
sta0654	Nov 03 2010	12:05:15	68 47.03 N	012 57.93 E	188
sta0658	Nov 03 2010	23:23:26	69 07.99 N	011 56.99 E	2912
sta0664	Nov 05 2010	4:22:22	70 00.04 N	005 59.98 E	3209
sta0667	Nov 07 2010	12:47:41	64 40.01 N	000 00.19 W	2744
sta0668	Nov 07 2010	17:17:23	64 22.16 N	000 43.46 E	2560
sta0677	Nov 08 2010	20:25:10	62 53.93 N	004 02.95 E	748
